# Ureteral metastasis incidentally found from primary breast cancer: A case report and review of the literature

**DOI:** 10.1016/j.eucr.2025.103003

**Published:** 2025-03-05

**Authors:** Matthew Satariano, Vivian Wong, Sarah Sweigert

**Affiliations:** a Northeast Ohio Medical University, USA; b The Ohio State University Wexner Medical Center Department of Urology, USA

**Keywords:** Ureteral mass, Breast cancer metastasis

## Abstract

Breast cancer metastasis to the ureter has been rarely reported and is typically diagnosed by autopsy or radiographically rather than by pathological inspection. We present the rare case of a 60-year-old female in which computed tomography urography imaging during nephrolithiasis revealed an incidental ureteral mass. Pathology workup following mass biopsy was estrogen receptor positive, progesterone receptor negative, and HER2 negative. The patient was ultimately diagnosed with metastatic lobular carcinoma of breast origin based on pathology from the axillary lymph node tissue. This case adds to the body of literature of a rare presentation of ureteral involvement from primary breast cancer.

## 1 Introduction

Ureteral involvement of metastases typically results from prostate, bladder, colorectal, and lymphoma cancers.^1^ Breast cancer metastasis to the ureter has been rarely reported and is typically diagnosed by autopsy or radiographically rather than by pathological inspection. Less than ten cases have been reported of primary breast cancer metastasizing to the ureters in the twenty-first century.^2^ Breast cancer is the most common malignancy in adult females and typically metastasizes to the brain, lungs, liver, and bones.^2^ Metastases to the ureter typically present with flank pain, oliguria, or hematuria and usually involves the lower ureteral segment.^2^ Given the rarity of ureteral metastases from primary breast cancer, we present a case report of this rare presentation and the work up.

## 2 Case report

**Fig. 1:**
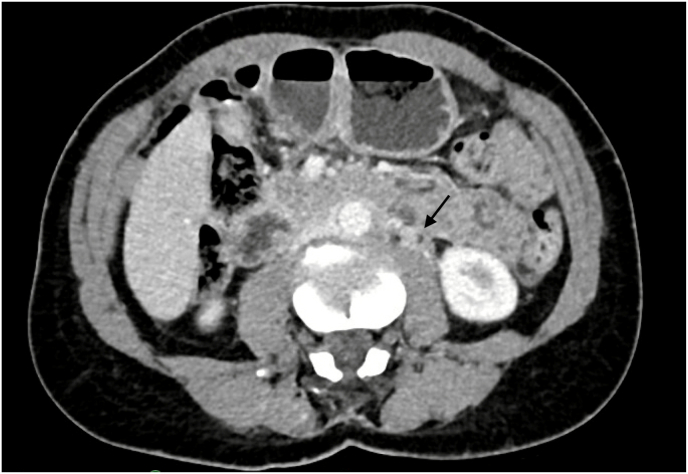
CT Urogram with arrow pointing to urothelial thickening in proximal ureter.

A 60-year-old female presented to the urology office with a history of recurrent nephrolithiasis to establish care. Surveillance renal ultrasound was obtained which showed left hydronephrosis so a computed tomography urography (CTU) was performed. This revealed urothelial thickening and abnormal enhancement in the left renal pelvis and in the left renal collecting system which extended along the proximal to mid left ureter (Fig. 1). The CTU also noted ill-defined soft tissue thickening and enhancement in the retroperitoneum and in the periaortic region especially on the left side with suspected confluent lymph nodes which appeared to encase segments of the proximal ureters and enlarged external iliac node. Given the unusual left-sided upper tract enhancement and left hydronephrosis, ureteroscopy in the operating room was performed for direct visualization which revealed a distal ureteral neoplasm. The lesion seen did not appear consistent with typical urothelial carcinoma. Left upper tract biopsy performed at this time revealed scant, detached, denuded fragment of tissue with infiltrating carcinoma with signet ring cell/plasmacytoid features. Intra-operatively, vaginal speculum exam revealed a thickened white anterior cervix which was biopsied and resulted as rare, atypical cells, indefinite for carcinoma. Cytology was negative for high grade urothelial carcinoma. Immunohistochemical stains were positive for AE1/3, CK7, GATA3 and negative for CK20, p63, CD163, estrogen receptor, progesterone receptor, and HER2.

**Fig. 2:**
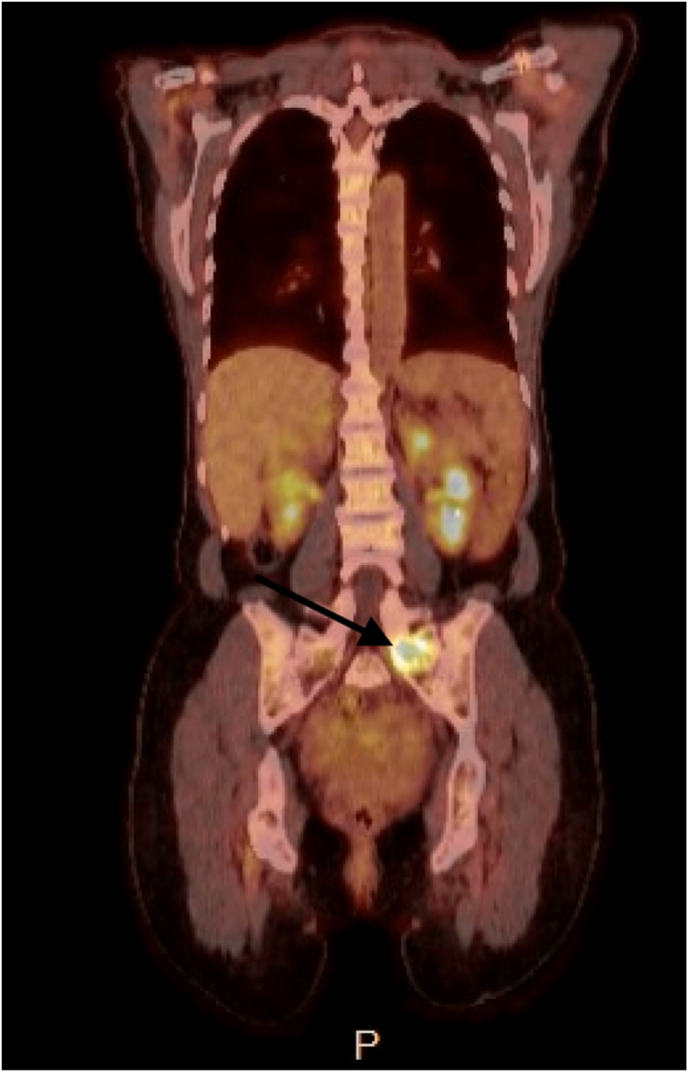
18-F-FDG PET/CT imaging with arrow pointing to FDG uptake in left pelvic sidewall lesion.

Further biopsy of enlarged left external iliac node revealed tumors cells strongly and diffusively positive for pancytokeratin-AE1/3, CK7, GATA3, and negative for CK20, thyroid transcription factor-1 (TTF-1), PAX8, CDX2, p40, and SOX10. Additional pathological investigation revealed the specimen was positive for the estrogen receptor (90 %, strong intensity), negative for the progesterone receptor, and negative for HER2 (Score 1+). Further molecular testing was then performed raising concern for metastatic cancer of breast origin. Right breast mammogram and bilateral magnetic resonance imaging (MRI) breast without suspicion for malignancy. Enlarged left axillary lymph nodes seen on MRI breast. Positron emission tomography (PET) scan with prominent retroperitoneal lymph nodes, adnexal and pelvic masses (Fig. 2). Biopsy of left axillary lymph nodes was performed. The patient was ultimately diagnosed with metastatic lobular carcinoma of breast origin based on pathology from the axillary lymph node tissue. The patient is established with oncology and currently on ribociclib and anastrozole therapy.

## 3 Discussion

Ureteral metastasis from primary breast cancer is extremely rare with only a handful of case reports in the literature. One study from 1969 analyzed 215 autopsies of patients with breast cancer and identified ureteral metastases in 42 (19.5 %) individuals.^3^

It is important to consider the pathological features of the case report. The focal signet ring cell features in the first biopsy were suggestive of urothelial carcinoma, plasmacytoid variant, although a primary breast carcinoma was still in the differential. On further biopsy analysis, estrogen receptor positivity was identified which helped make the final diagnosis of breast cancer. Although literature is scarce regarding the common pathological features of ureteral metastases from primary breast cancer, it was most commonly reported as estrogen receptor (ER) (+), progesterone receptor (+), and HER2 (−).^4^ One case report from 2020 detailed a human epidermal receptor 2 (HER2)-enriched breast cancer that metastasized to the left proximal ureter.^2^ Thus, our case report is one of the only with a ureteral metastases with an ER (+), progesterone receptor (−), and HER2 (−) presentation.

Some literature suggests trying to distinguish primary ureteral cancers from ureteral metastases. One case report proposed utilization of fluorine-18-fluorodeoxyglucose positron emission tomography (18F-FDG PET)/computed tomography (CT) to identify a ureteral involvement from a primary breast tumor.^5^ The authors describe ureteral metastases typically have no substantial change in ureteral contour, a long segmental lesion, and limited thickening of the ureteral wall on CT compared to primary ureteral cancers. However, literature is inconclusive on whether 18-F-FDG PET/CT imaging in urologic oncology due to its excretion of FDG in the urine and limited uptake.^6^

## 4 Conclusion

Ureteral metastasis from primary breast cancer is rare with only a handful of case reports in the literature. We present the case of a 60-year-old female in which ureteral metastasis was incidentally found in which primary breast cancer was later diagnosed after extensive pathological workup.

## CRediT authorship contribution statement

**Matthew Satariano:** Writing – original draft, Writing – review & editing. **Vivian Wong:** Writing – original draft, Writing – review & editing. **Sarah Sweigert:** Supervision, Writing – review & editing.

## Consent

The proper consent was obtained for this research.

## Funding

This research did not receive any specific grant from funding agencies in the public, commercial, or not-for-profit sectors.

## Conflict of interest statement

There are no conflicts of interest.
